# Five rules for friendly rivalry in direct reciprocity

**DOI:** 10.1038/s41598-020-73855-x

**Published:** 2020-10-09

**Authors:** Yohsuke Murase, Seung Ki Baek

**Affiliations:** 1 RIKEN Center for Computational Science, Kobe, Hyogo 650-0047 Japan; 2 Department of Physics, Pukyong National University, Busan, 48513 Korea

**Keywords:** Social evolution, Computational science

## Abstract

Direct reciprocity is one of the key mechanisms accounting for cooperation in our social life. According to recent understanding, most of classical strategies for direct reciprocity fall into one of two classes, ‘partners’ or ‘rivals’. A ‘partner’ is a generous strategy achieving mutual cooperation, and a ‘rival’ never lets the co-player become better off. They have different working conditions: For example, partners show good performance in a large population, whereas rivals do in head-to-head matches. By means of exhaustive enumeration, we demonstrate the existence of strategies that act as both partners and rivals. Among them, we focus on a human-interpretable strategy, named ‘CAPRI’ after its five characteristic ingredients, i.e., cooperate, accept, punish, recover, and defect otherwise. Our evolutionary simulation shows excellent performance of CAPRI in a broad range of environmental conditions.

## Introduction

Theory of repeated games is one of the most fundamental mathematical frameworks that has long been studied for understanding how and why cooperation emerges in human and biological communities. Even when cooperation cannot be a solution of a one-shot game, repetition can enforce cooperation between the players by taking into account the possibility of future encounters. A spectacular example is the prisoner’s dilemma (PD) game: It describes a social dilemma between two players, say, Alice and Bob, in which each player has two options ‘cooperation’ (*c*) and ‘defection’ (*d*). The payoff matrix for the PD game is defined as follows:1where each entry shows (Alice’s payoff, Bob’s payoff) with $T>R>P>S$ and $2R > T+S$. If the game is played once, mutual defection is the only equilibrium because Alice maximizes her payoff by defecting no matter what Bob does. However, if the game is repeated with sufficiently high probability, cooperation becomes a feasible solution because the players have a strategic option that they can reward cooperators by cooperating and/or they can punish defectors by defecting in subsequent rounds (see, e.g., Table 1). This is known as direct reciprocity, one of the most well-known mechanisms for the evolution of cooperation^1^.

Through a series of studies, recent understanding of direct reciprocity proposes that most of well-known strategies act either as partners or as rivals^2,3^. Partner strategies are also called ‘good strategies’^4,5^, and rival strategies have been described as ‘unbeatable’^6^, ‘competitive’^2^, or ‘defensible’^7,8^. Derived from our everyday language, the ‘partner’ and ‘rival’ are defined as follows. As a partner, Alice aims at sharing the mutual cooperation payoff *R* with her co-player Bob. However, when Bob defects from cooperation, Alice will punish Bob so that his payoff becomes less than *R*. In other words, for Alice’s strategy to be a partner, we need the following two conditions: First, $\pi _{A} = \pi _{B} = R$ when Bob applies the same strategy as Alice’s, where $\pi _{A}$ and $\pi _{B}$ represent the long-term average payoffs of Alice and Bob, respectively. Second, when $\pi _{A}$ is less than *R* because of Bob’s defection from mutual cooperation, $\pi _{B}$ must also be smaller than *R*, whatever Bob takes as his strategy. It means that one of the best responses against a partner strategy is choosing the same partner strategy so that they form a Nash equilibrium. If a player uses a rival strategy, on the other hand, the player aims at a payoff higher than or equal to the co-player’s regardless of the co-player’s strategy. Thus, as long as Alice is a rival, it is guaranteed that $\pi _{A} \ge \pi _{B}$. Note that these two definitions impose no restriction on Bob’s strategy, which means that the inequalities are unaffected even if Bob remembers arbitrarily many previous rounds.

Which of these two traits is favoured by selection depends on environmental conditions, such as the population size *N* and the elementary payoffs *R*, *T*, *S*, and *P*. For instance, a large population tends to adopt partner strategies when *R* is high enough. A natural question would be on the possibility that a single strategy is *both* a partner and a rival simultaneously: The point is not to gain an extortionate payoff from the co-player in the sense of the zero-determinant (ZD) strategies^9^ but to provide an incentive to form mutual cooperation. Let us call such a strategy a ‘friendly rival’ hereafter. Tit-for-tat (TFT) or Trigger strategies can be friendly rivals in an ideal condition that the players are free from implementation error due to “trembling hands”. However, this is not the case in a more realistic situation in which actions can be misimplemented with probability $e > 0$. Here, the apparent contradiction between the notions of a partner and a rival is seen as the most acute form. That is, Alice must forgive Bob’s erroneous defection to be a partner *and* punish his malicious defection to be a rival, without knowing Bob’s intention. This is the crux of the matter in relationships.

In this work, by means of massive supercomputing, we show that a tiny fraction of friendly rival strategies exist among deterministic memory-three strategies for the iterated PD game without future discounting. Differently from earlier studies^9–17^, our strategies are deterministic ones, which makes each of them easy to implement as a behavioural guideline as well as a public policy without any randomization device^18^. In particular, we focus on one of the friendly rivals, named CAPRI, because it can be described in plain language, which implies great potential importance in understanding and guiding human behaviour. We also argue that our friendly rivals exhibit evolutionary robustness^13^ for any population size and for any benefit-to-cost ratio. This property is demonstrated by evolutionary simulation in which CAPRI overwhelms other strategies under a variety of environmental parameters.

**Table 1:** Description of well-known strategies in the iterated PD game. Whenever possible, each strategy is represented as a tuple of five probabilities, i.e., $(p_0, p_{R}, p_{S}, p_{T}, p_{P})$, where $p_0$ means the probability to cooperate in the first round, and $p_{\beta }$ means the probability to cooperate after obtaining payoff $\beta$ in the previous round (see Eq. 1). Here, a zero-determinant (ZD) strategy has a positive parameter $\phi$, and its other parameter $\eta$ lies in the unit interval^9,13,19^.

Strategy	Description
AllC	(1, 1, 1, 1, 1)
AllD	(0, 0, 0, 0, 0)
Tit-for-tat (TFT)	(1, 1, 0, 1, 0)
Generous TFT	(1, 1, *q*, 1, *q*) with $0<q<1$
Tit-for-two-tats (TF2T)	Defect if the co-player defected in the previous two rounds.
Win-Stay-Lose-Shift (WSLS)	(1, 1, 0, 0, 1)
generous ZD	$(1, 1, 1-\phi [(1-\eta )(S-R)+T-S], \phi [(1-\eta )(R-T)+T-S], \phi (1-\eta )(R-P))$
extortionate ZD	$(0, 1-\phi (1-\eta )(R-P), 1-\phi [(1-\eta )(S-P)+T-S], \phi [(1-\eta )(P-T)+T-S], 0)$
Trigger	Defect if defection has ever been observed.

## Methods

Despite the fundamental importance of memory in direct reciprocity, combinatorial explosion has been a major obstacle in understanding the memory effects on cooperation: Let us consider deterministic strategies with memory length *m*, which means that each of them chooses an action between *c* and *d* as a function of the *m* previous rounds. The number of such memory-*m* strategies expands as $N=2^{2^{2m}}$, which means $N_{m=1} = 16$, $N_{m=2} = 65536$, and $N_{m=3} \approx 1.84\times 10^{19}$. The number of combinations of these strategies grows even more drastically, which renders typical evolutionary simulation incapable of exploring the full strategy space. Here, we take an axiomatic approach^7,8,20^ to find friendly rivals. That is, we search for strategies that satisfy certain predetermined criteria, and the computation time for checking those criteria scales as *O*(*N*) instead of $O(N^2)$ or greater.

**Figure 1:**
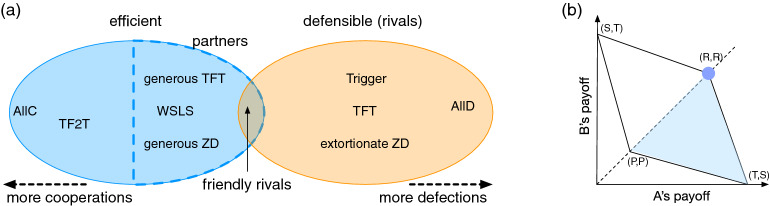
(Left) A schematic diagram of the strategy space. Strategies that tend to cooperate (defect) are shown on the left (right). The blue ellipse represents a set of efficient strategies, which are cooperative to sustain mutual cooperation, and its subset of partner strategies is denoted by the dashed blue curve. On the other hand, the red ellipse represents a set of defensible strategies, which often defect to defend themselves from malicious co-players. In general, their intersection is small. When $m = 2$, for instance, the sizes of efficient and defensible sets are 7639 and 2144, respectively, whereas the intersection contains only eight strategies. (Right) The diamond depicts the region of possible average payoffs for Alice and Bob. The blue triangle shows the feasibility region when Alice uses a defensible strategy. If Alice and Bob both use the same strategy satisfying efficiency, they will reach (*R*, *R*) (the blue dot).

More specifically, we begin with the following two criteria^7,8^:

1. Efficiency: Mutual cooperation is achieved with probability one as error probability $e \rightarrow 0^+$, if both Alice and Bob use this strategy.
2. Defensibility: If Alice uses this strategy, she will never be outperformed by Bob when $e=0$ regardless of initial actions. This is a sufficient condition for being a rival, i.e., $\lim _{e\rightarrow 0^+}(\pi _{A} - \pi _{B}) \ge 0$.

The efficiency criterion requires a strategy to establish cooperation in the presence of small *e* when both the players adopt this strategy. This criterion is satisfied by many generous strategies such as unconditional cooperation (AllC), generous TFT (GTFT), Win-Stay-Lose-Shift (WSLS) and Tit-for-two-tats (TF2T). Partner strategies constitute a sub-class of efficient ones by limiting the co-player’s payoff to be less than or equal to *R* regardless of the co-player’s payoff^2,3,5^. On the other hand, a defensible strategy must ensure that the player’s long-time average payoff will be no less than that of the co-player who may use *any* possible strategy, and this idea is equivalent to the notion of a ‘rival strategy’^2,3^. Defensible strategies include unconditional defection (AllD), Trigger, TFT, and extortionate ZD strategies. Figure 1a schematically shows how these two criteria narrow down the list of strategies to consider. The overlap of efficient and defensible strategies means a set of friendly rivals because it is a subset of partner strategies. It assigns the most strict limitation on the co-player’s payoff among the partner strategies as shown in Fig. 1b. Indeed, the overlap region between these two criteria is extremely tiny: It is pure impossibility for $m=1$, and we find only 8 strategies out of $N=65536$ for $m=2$.

To further narrow down the list of strategies, we impose the third criterion^7,8^:

3. Distinguishability: The strategy has a strictly higher payoff than the co-player’s when its strategy is AllC in the small-error limit, i.e., $\lim _{e\rightarrow 0^{+}}(\pi _{A} - \pi _{\mathrm{AllC}}) > 0$.

This requirement originates from evolutionary game theory: If this criterion is violated, the number of AllC players may increase due to neutral drift, which eventually makes the population vulnerable to invasion of defectors such as AllD. We check these criteria for each strategy by representing it as a graph and analysing its topological properties (see Supplementary Methods S1 at the end of this manuscript). If a strategy satisfies all those three criteria, it will be called ‘successful’.

Among deterministic memory-two strategies, it is known that only four strategies satisfy these three criteria^7^. They have minor differences from each other, and one of them is called TFT-ATFT, which is a combination of TFT and anti-tit-for-tat (ATFT). It usually behaves as TFT, but it takes the opposite moves after mistakenly defecting from mutual cooperation. Similar analysis has been conducted for the three-person public-goods (PG) game: At least 256 successful strategies exist when $m=3$, whereas no such solution exists when $m<3$^8^. It has also been shown that a friendly rival strategy must have $m \ge n$ for the general *n*-person PG game, although such a strategy for $n > 3$ is yet to be found. These results suggest that a novel class of strategies may appear as the memory length exceeds a certain threshold.

For memory-three strategies, we have obtained an exhaustive list of successful strategies by massive supercomputing (see Supplementary Methods S1 at the end of this manuscript). The efficiency and defensibility criteria find 7, 018, 265, 885, 034 friendly rivals out of $N_{m=3} = 2^{64} \approx 1.84 \times 10^{19}$ strategies. If the distinguishability criterion is additionally imposed, 4, 261, 844, 305, 281 strategies are found. There are four actions commonly prescribed by all these successful strategies: Let $A_t$ and $B_t$ denote Alice’s and Bob’s actions at time *t*, respectively. When their memory states are $(A_{t-3}A_{t-2}A_{t-1},B_{t-3}B_{t-2}B_{t-1}) = (ccc,ccc)$, (*ccc*, *ddd*), (*cdd*, *ddd*), and (*ddd*, *ddd*), all the successful strategies tell Alice to choose *c*, *d*, *d*, and *d*, respectively. The first one is absolutely required to maintain mutual cooperation. The latter three are needed to satisfy the defensibility criterion: If *c* was prescribed at any of these states, Alice would be exploited by Bob’s continual defection.

**Table 2:** Recovery paths to mutual cooperation for the memory-three successful strategies. Only the most common five patterns are shown in this table.

Action sequence	# of strategies
$$\begin{array}{cccccc}\dots &{} c &{} c &{} d &{} c &{} \dots \\ \dots &{} c &{} d &{} c &{} c &{} \dots \end{array}$$	$$\begin{array}{r} 905,772,524,235 \\ (21.3\%) \end{array}$$
$$\begin{array}{cccccccc}\dots &{} c &{} c &{} d &{} d &{} c &{} \dots \\ \dots &{} c &{} d &{} c &{} d &{} c &{} \dots \end{array}$$	$$\begin{array}{r} 522,061,013,252 \\ (12.2\%) \end{array}$$
$$\begin{array}{ccccccc}\dots &{} c &{} c &{} d &{} d &{} c &{} \dots \\ \dots &{} c &{} d &{} d &{} c &{} c &{} \dots \end{array}$$	$$\begin{array}{r} 437,671,509,356 \\ (10.3\%) \end{array}$$
$$\begin{array}{cccccccc}\dots &{} c &{} c &{} d &{} c &{} d &{} c &{} \dots \\ \dots &{} c &{} d &{} c &{} c &{} d &{} c &{} \dots \end{array}$$	$$\begin{array}{r} 409,458,612,318 \\ (9.6\%) \end{array}$$
$$\begin{array}{ccccccccc}\dots &{} c &{} c &{} d &{} c &{} d &{} d &{} c &{} \dots \\ \dots &{} c &{} d &{} c &{} c &{} d &{} d &{} c &{} \dots \end{array}$$	$$\begin{array}{r} 227,113,898,468 \\ (5.3\%) \end{array}$$
$$\begin{array}{cccccccc}\dots &{} c &{} c &{} d &{} d &{} d &{} c &{} \dots \\ \dots &{} c &{} d &{} c &{} c &{} d &{} c &{} \dots \end{array}$$	$$\begin{array}{r} 184,052,002,852 \\ (4.3\%) \end{array}$$

The upper and lower rows represent the sequences of actions taken by Alice and Bob, respectively, when Bob defected from mutual cooperation by error. The right column shows the number of strategies having each pattern, as well as its fraction with respect to the total number of successful strategies.

Except for these four prescriptions, we see a wide variety of patterns. For example, let us assume that both Alice and Bob adopt one of these strategies. When Bob defects by error, they must follow a recovery path from state (*ccc*, *ccd*) to (*ccc*, *ccc*). We find 839 different patterns from our successful strategies (Table 2). The most common one is also the shortest, in which only two time steps are needed to recover mutual cooperation. It cannot be shorter because Alice must defect at least once to assure defensibility. It is even shorter than that of TFT-ATFT, which is identical to the third entry of Table 2. This finding disproves a speculation that friendly rivals are limited to variants of TFT even if $m>2$^7^. This shortest recovery path is possible only when $m \ge 3$, indicating a pivotal role of memory length in direct reciprocity.

## Results

### CAPRI strategy

The shortest recovery path in Table 2 shows that Bob can recover his own mistake simply by accepting Alice’s punishment provided that he has $m=3$. Among the strategies using this recovery pattern, we have discovered a strategy which is easy to interpret, named ‘CAPRI’, after the first letters of its five constitutive rules listed below:

1. Cooperate at mutual cooperation. This rule prescribes *c* at (*ccc*, *ccc*).
2. Accept punishment when you mistakenly defected from mutual cooperation. This rule prescribes *c* at (*ccd*, *ccc*), (*cdc*, *ccd*), (*dcc*, *cdc*), and (*ccc*, *dcc*).
3. Punish your co-player by defecting once when he defected from mutual cooperation. This rule prescribes *d* at (*ccc*, *ccd*), and then *c* at (*ccd*, *cdc*), (*cdc*, *dcc*), and (*dcc*, *ccc*).
4. Recover cooperation when you or your co-player cooperated at mutual defection. This rule prescribes *c* at (*ddd*, *ddc*), (*ddc*, *dcc*), (*dcc*, *ccc*), (*ddc*, *ddd*), (*dcc*, *ddc*), (*ccc*, *dcc*), (*ddc*, *ddc*), and (*dcc*, *dcc*).
5. In all the other cases, defect.

**Figure 2:**
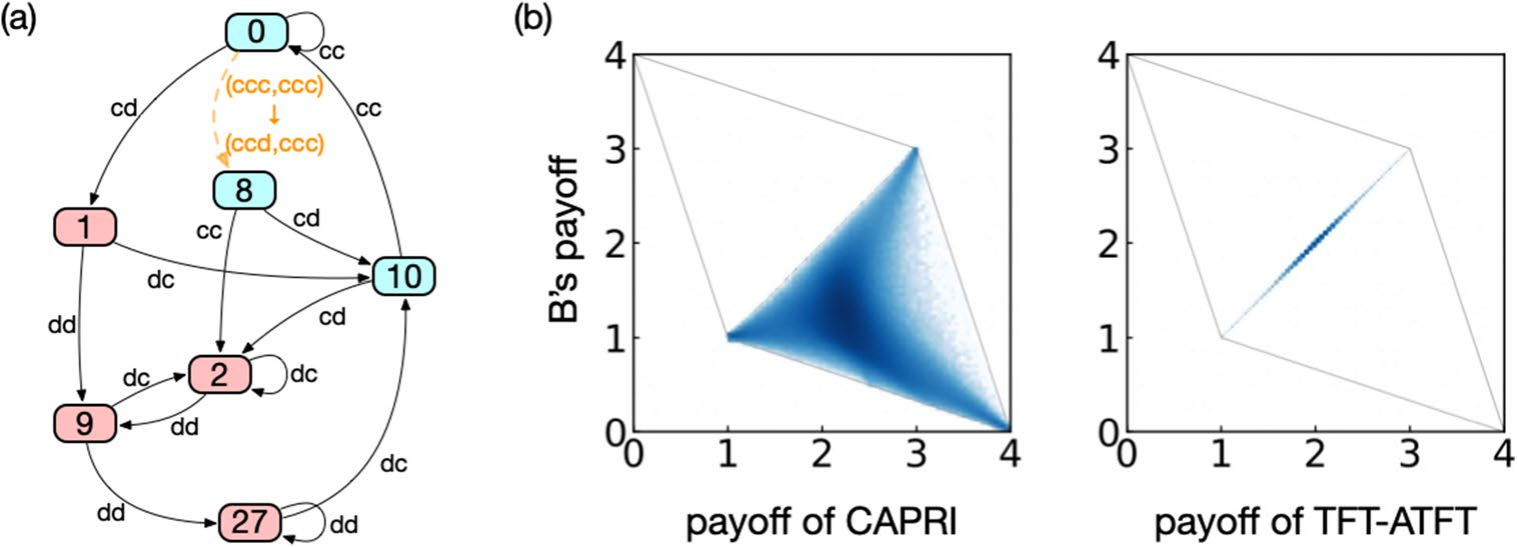
(**a**) Automaton representation of CAPRI. Its prescribed actions are denoted by the node colours (blue for *c* and red for *d*). The labels on the edges indicate the players’ actions. The transition caused by erroneous defection at mutual cooperation (‘0’) is depicted by an orange dashed arrow. (**b**) Distribution of payoffs when Alice’s strategy is CAPRI (left) or TFT-ATFT (right), whereas Bob adopts one of probabilistic memory-three strategies uniformly at random. The elementary payoffs are $(R,T,S,P)=(3,4,0,1)$.

The first rule is clearly needed for efficiency. In addition, mutual cooperation must be robust against one-bit error, i.e., occurring with probability of *O*(*e*), when both Alice and Bob use this strategy. This property is provided by the second and the third rules. In addition, for this strategy to be efficient, the players must be able to escape from mutual defection through one-bit error so that the stationary probability distribution does not accumulate at mutual defection, which is handled by the fourth rule. Note that these four rules for efficiency do not necessarily violate defensibility when $m>2$, as we have already seen in Table 2. Actually, due to the fifth rule, both efficiency and defensibility are satisfied by CAPRI. The action table and its minimized automaton representation^21^ are given in Table 3 and Fig. 2a, respectively. The self-loop via *dc* at state ‘2’ in Fig. 2a proves that this strategy also satisfies distinguishability.

CAPRI requires $m=3$ because otherwise it violates defensibility: If CAPRI were a memory-two strategy, $(cd,dc)\rightarrow c$ and $(dc,cd)\rightarrow c$ must be prescribed to recover from error. However, with these prescriptions, Bob can repeatedly exploit Alice by using the following sequence:

2 $$\begin{aligned} \begin{matrix}\dots &{} c &{} c &{} d &{} c &{} c &{} \dots \\ \dots &{} c &{} d &{} c &{} d &{} c &{} \dots . \end{matrix} \end{aligned}$$

TFT-ATFT and its variants are the only friendly rivals when $m<3$. Compared with TFT-ATFT, CAPRI is closer to Grim trigger (GT) rather than to TFT. Alice keeps cooperating as long as Bob cooperates, but she switches to defection, as prescribed by the fifth rule, when Bob does not conform to her expectation. Due to the similarity of CAPRI to GT, it also outperforms a wider spectrum of strategies than TFT-ATFT. Figure 2b shows the distribution of payoffs of the two players when Alice’s strategy is CAPRI, and Bob’s strategy is sampled from the 64-dimensional unit hypercube of memory-three probabilistic strategies. Alice’s payoff is strictly higher than Bob’s in most of the samples. On the other hand, when Alice uses TFT-ATFT, payoffs are mostly sampled on the diagonal because it is based on TFT, which equalizes the players’ payoffs. However, we also note that CAPRI is significantly different from GT in two ways. First, CAPRI is error-tolerant: Even when Bob makes a mistake, Alice is ready to recover cooperation after Bob accepts punishment, as described in the second and the third rules. Second, whereas GT is characterized by its irreversibility, CAPRI lets the players escape from mutual defection according to the fourth rule.

**Table 3:** Action table of CAPRI.

$A_{t-3}A_{t-2}A_{t-1}$	$B_{t-3}B_{t-2}B_{t-1}$							
	*ccc*	*ccd*	*cdc*	*cdd*	*dcc*	*dcd*	*ddc*	*ddd*
*ccc*	$^1c$	$^3d$	*d*	*d*	$^{2,4}c$	*d*	*d*	*d*
*ccd*	$^2c$	*d*	$^3c$	*d*	*d*	*d*	*d*	*d*
*cdc*	*d*	$^2c$	*d*	*d*	$^3c$	*d*	*d*	*d*
*cdd*	*d*	*d*	*d*	*d*	*d*	*d*	*d*	*d*
*dcc*	$^{3,4}c$	*d*	$^2c$	*d*	$^4c$	*d*	$^4c$	*d*
*dcd*	*d*	*d*	*d*	*d*	*d*	*d*	*d*	*d*
*ddc*	*d*	*d*	*d*	*d*	$^4c$	*d*	$^4c$	$^4c$
*ddd*	*d*	*d*	*d*	*d*	*d*	*d*	$^4c$	*d*

The superscript on the upper left corner of each element indicates which rule is involved.

### Evolutionary simulation

**Figure 3:**
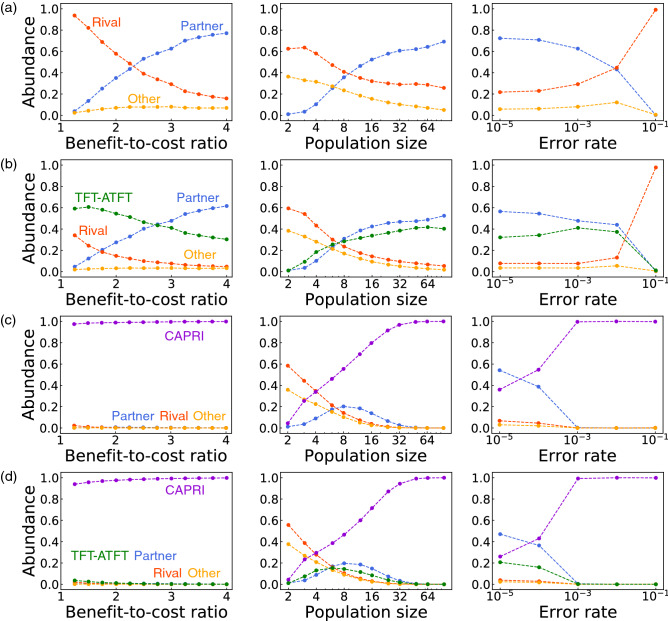
(**a**) Abundance of memory-one partners, rivals, and the other strategies. We consider a simplified version of the PD game, parametrized by *b*, the benefit to the co-player when a player cooperates with a unit cost. In terms of the elementary payoffs, this corresponds to $R = b-1$, $T=b$, $S=-1$, and $P=0$. The Moran process is simulated with selection strength $\sigma$ in a population of size *N*, where the product $N \sigma$ is fixed as 10. Three parameters (benefit-cost-ratio *b*, population size *N*, and error rate *e*) are varied one by one^3^. Their default values are $b = 3, N = 50, \text{ and }e = 10^{-3}$ unless otherwise stated. We also show the simulation results with (**b**) TFT-ATFT, (**c**) CAPRI, and (**d**) both TFT-ATFT and CAPRI, introduced with probability $\mu = 0.01$. These are average results over 10 Monte–Carlo runs.

Although defensibility assures that the player is never outperformed by the co-player, it does not necessarily guarantee success in evolutionary games, where everyone is pitted against every other in the population. For example, extortionate ZD strategies perform poorly in an evolutionary game^12,13,22^. In this section, we will check the performance of CAPRI in the evolutionary context.

When we consider performance of a strategy in an evolving population, the most famous measure of assessment is evolutionary stability (ES)^23^. Although conceptually useful, ES is too strong a condition, requiring that when a sufficient majority of population members apply the strategy, every other strategy is at a selective disadvantage. Evolutionary robustness has thus been introduced as a more practical notion of stability^13^: A strategy is called evolutionary robust if no other strategy has fixation probability greater than 1/*N*, which is the fixation probability of a neutral mutant. In other words, an evolutionary robust strategy cannot be selectively replaced by any mutant strategy. Evolutionary robustness of a strategy depends on the population size: Partner strategies have this property when *N* is large enough, whereas for rival strategies, it is when *N* is small^13^. Friendly rivals have the virtue of both: They keep evolutionary robustness regardless of *N*, as will be shown below.

As in the standard stochastic model^24^, let us consider a well-mixed population of size *N* in which selection follows an imitation process. At each discrete time step, a pair of players are chosen at random, and we will call their strategies *X* and *Y*, respectively. The probability for one of the players to replace her strategy *X* with *Y* is given as follows:

3 $$\begin{aligned} f_{x \rightarrow y} = \frac{1}{1 + \exp \left[ \sigma \left( s_x - s_y\right) \right] }, \end{aligned}$$

where $s_x$ and $s_y$ denote the average payoffs of *X* and *Y* against the entire population, respectively, and $\sigma$ is a parameter which denotes the strength of selection. In population dynamics, we assume that the mutation rate $\mu$ is low enough: That is, when a mutant strategy *X* appears in a resident population of *Y*, no other mutant will be introduced until *X* reaches fixation or goes extinct. The dynamics is formulated as a Moran process, under which the fixation probability of *X* is given in a closed form^13^:

4 $$\begin{aligned} \rho = \frac{1}{\sum _{i=0}^{N-1}\prod _{j=1}^{i} e^{\sigma \left[ (N-j-1)s_{yy} + js_{yx} - (N-j)s_{xy} - (j-1)s_{xx} \right] }}, \end{aligned}$$

where $s_{xy}$ denotes the long-term payoff of player *X* against player *Y*. Using Jensen’s inequality, we see that

5 $$\begin{aligned} \frac{1}{\rho }= & {} \sum _{i=0}^{N-1} e^{ \sigma i \left[ (2N-i-3)s_{yy} + (i+1)s_{yx} - (2N-i-1)s_{xy} -(i-1)s_{xx} \right] /2 } \end{aligned}$$

6 $$\begin{aligned}\ge & {} N e^{ \sigma (N-1) \left[ (N-2)(s_{yy}-s_{xx}) + (N+1)(s_{yx}-s_{xy}) + (N-2)(s_{yy} - s_{xy}) \right] /6 }. \end{aligned}$$

When *Y* is a partner strategy, it satisfies $s_{yy} \ge s_{xy}$ and $s_{yy} \ge s_{xx}$. When *Y* is also a rival strategy, it has another inequality, $s_{yx} \ge s_{xy}$. Therefore, the fixation probability of an arbitrary mutant $\rho \le 1/N$ regardless of *N* and $\sigma$.

We have conducted evolutionary simulation to assess the performance of friendly rivals. First, we run simulation without CAPRI and TFT-ATFT. This simulation adopts the setting of a recent study^3^ and serves as a baseline of performance. A mutant strategy is restricted to reactive memory-one strategies, according to which the player’s action depends only on the co-player’s last action. The reactive strategies are characterized by a pair of probabilities ($p_{c}$,$p_{d}$), where $p_\alpha$ denotes the probability to cooperate when the co-player’s last move was $\alpha$. Rival strategies are represented by $p_d = 0$, and partners are by $p_c = 1$ and $p_d < p_d^{*}$, where $p_d^{*} \equiv \min \{1-(T-R)/(R-S),(R-P)/(T-P)\}$. Mutant strategies may be randomly drawn from $[0,1] \times [0,1]$, but we have discretized the unit square in a way that each $p_\alpha$ takes a value from $\{0, 1/10, 2/10, \dots , 9/10, 1\}$. We have run the simulation until mutants are introduced $10^7$ times, and measured how frequently partner or rival strategies are observed. As shown in Fig. 3a, evolutionary performance of strategies depends on environmental parameters^3,13,14^. Rival strategies have higher abundance when the benefit-to-cost ratio is low, population size *N* is small, and error rate *e* is high. Otherwise, partner strategies are favoured.

Let us now assume that a mutant can also take TFT-ATFT in addition to the reactive memory-one strategies. Figure 3b shows that TFT-ATFT occupies significant fractions across a broad range of parameters. The situation changes even more remarkably when CAPRI is introduced instead of TFT-ATFT. As seen in Fig. 3c, CAPRI overwhelms the other strategies for almost the entire parameter ranges. The low abundance at $N=2$ or $e=10^{-5}$ does not contradict with the evolutionary robustness of CAPRI because it is still higher than the abundance of a neutral mutant. Although the abundance of partners is higher than CAPRI when $e=10^{-5}$, the reason is that it is an aggregate over many partner strategies. If we compare each single strategy, CAPRI is still the most abundant one for the entire range of *e*. The qualitative picture remains the same even if we choose a different value of $\sigma$, and CAPRI tends to be more favoured as $\sigma$ increases. Furthermore, by comparing Fig. 3b,c, we see that CAPRI shows better performance than TFT-ATFT. The evolutionary advantage of CAPRI over TFT-ATFT is directly observed in Fig. 3d), where both CAPRI and TFT-ATFT are introduced into the population. As we have seen in Fig. 2b, it tends to earn strictly higher payoffs against various types of co-players, whereas TFT-ATFT, based on TFT, aims to equalize the payoffs except when it encounters naive cooperators. This observation shows a considerable amount of diversity even among evolutionary robust strategies^25^.

## Summary

To summarize, we have investigated the possibility to act as both a partner and a rival in the repeated PD game without future discounting. By thoroughly exploring a huge number of strategies with $m=3$, we have found that it is indeed possible in various ways. The resulting friendly rivalry directly implies evolutionary robustness for any population size, benefit-to-cost ratio, and selection strength. We observe its success even when *e* is of a considerable size (Fig. 3). It is also worth noting that a friendly rival can publicly announce its strategy because it is guaranteed not to be outperformed regardless of the co-player’s prior knowledge. Rather, it is desirable that the strategy should be made public because the co-player can be advised to adopt the same strategy by knowing it from the beginning to maximize its payoff. The resulting mutual cooperation is a Nash equilibrium. The deterministic nature offers additional advantages because the player can implement the strategy without any randomization device. Moreover, even if uncertainty exists in the cost and benefit of cooperation, a friendly rival retains its power because it is independent of (*R*, *T*, *S*, *P*). This is a distinct feature compared to the ZD strategies, whose cooperation probabilities have to be calculated from the elementary payoffs. Furthermore, the results are independent of the specific payoff ordering $T>R>P>S$ of the PD. These are valid as long as mutual cooperation is socially optimal ($R>P$ and $2R > T+S$) and exploiting the other’s cooperation pays better than being exploited ($T>S$). This condition includes other well-known social dilemma, such as the snowdrift game (with $T>R>S>P$) and the stag-hunt game (with $R>T>P>S$).

This work has focused on one of friendly rivals, named CAPRI. We speculate that it is close to the optimal one in several respects: First, it recovers mutual cooperation from erroneous defection in the shortest time. Second, it outperforms a wide range of strategies. Furthermore, its simplicity is almost unparalleled among friendly rivals discovered in this study. CAPRI is explained by a handful of intuitively plausible rules, and such simplicity greatly enhances its practical applicability because the required cognitive load will be low when we humans play the strategy^11,26,27^. It is an interesting research question whether this statement can be verified experimentally.

In particular, we would like to stress the importance of memory length in theory and experiment, considering that much research attention has been paid to the study of memory-one strategies^2,5,13,14,28–30^. Besides the combinatorial explosion of strategic possibilities, one can argue that a memory-one strategy, if properly designed, can unilaterally control the co-player’s payoff even when the co-player has longer memory^9^. It has also been shown that $m=1$ is enough for evolutionary robustness against mutants with longer memory^13^. However, the payoff that a strategy receives against itself may depend on its own memory capacity^13,25^, and this is the reason that a friendly rival is feasible when $m>1$. We can gain some important strategic insight only by moving beyond $m=1$. Related to the above point, one of important open problems is how to design a friendly-rival strategy for multi-player games. Little is known of the relationship between a solution of an *n*-person and that of an $(n-1)$-person game of the same kind. For example, it is known that TFT-ATFT for the PD game^7^ is not directly applicable to the three-person PG game^8^. We nevertheless hope that the five rules of CAPRI may be more easily generalized to the *n*-person game, considering that its working mechanism seems more comprehensible than that of TFT-ATFT to the human mind.

In a broader context, although ‘friendly rivalry’ sounds self-contradictory, the term captures a crucial aspect of social interaction when it goes in a productive way: Rivalry is certainly ubiquitous between artists, sports teams, firms, research groups, or neighbouring countries^31–34^. At the same time, they are subject to repeated interaction, whereby they eventually become friends, colleagues, or business partners to each other. Our finding suggests that such a seemingly unstable relationship can readily be sustained just by following a few simple rules: Cooperate if everyone does, accept punishment for your mistake, punish defection, recover cooperation if you find a chance, but in all the other cases, just take care of yourself. These seem to be the constituent elements for such a sophisticated compound of rivalry and partnership.

## Supplementary information

Supplementary material 1

## Data Availability

The source code for this study is available at https://github.com/yohm/sim_exhaustive_m3_PDgame.
