# Supplementary material for: Five rules for friendly rivalry in direct reciprocity
Source: Sci Rep. 2020 Oct 9;10:16904. doi: 10.1038/s41598-020-73855-x (PMC7547665; doi:10.1038/s41598-020-73855-x)
Supplement: Supplementary file 1 — Supplementary material 1 [file 41598_2020_73855_MOESM1_ESM.pdf]

Supplementary Material

Five rules for friendly rivalry in direct reciprocity

Yohsuke Murase
RIKEN Center for Computational Science, Kobe, Hyogo 650-0047, Japan

Seung Ki Baek
Department of Physics, Pukyong National University, Busan 48513, Korea

September 28, 2020

Supplementary Methods

We wish to examine the strategy space of $m = 3$, but it is impossible to enumerate all the memory-three strategies by a naive brute-force method even if we use a cutting-edge supercomputer because their total number is as large as $2^{2^{nm}} = 2^{64} \approx 2 \times 10^{19}$. To overcome this difficulty, we have developed graph-theoretic algorithms to judge defensibility, efficiency, and distinguishability. In the following, we explain the algorithm in three steps: First, we present basic ideas to judge the three criteria for a single strategy. Second, we show how this can be done for a set of strategies simultaneously. Third, we apply these algorithms to enumerate all successful strategies comprehensively in the memory-three strategy space. A C++ source code is available under an open-source license at `https://github.com/yohm/sim_exhaustive_m3_PDgame`.

Judging the criteria for a single strategy

Let us consider two players A and B in the iterated PD game. Player A's action at time t is denoted as A_t, and B_t is defined likewise. When $m = 3$, we have 64 different history profiles, $(A_{t-3}A_{t-2}A_{t-1}, B_{t-3}B_{t-2}B_{t-1}) = (ccc, ccc)$, (ccc, ccd), (ccc, cdc), $\ldots (ddd, ddd)$. These profiles can also be represented as 0, 1, 2, $\ldots$ 63 in binary. Consider a directed graph whose nodes represent the history profiles and whose links represent transition among them as prescribed by S_A and S_B, where S_A and S_B are the strategies of the players A and B, respectively. Such a graph will be called a transition graph in general. Due to the deterministic property of S_A and S_B, each node has one outgoing link in the absence of error, so the total number of links is also 64. We will denote this graph as $g(S_A, S_B)$.

We may also consider another transition graph for the case where B's actions are left undetermined whereas A's strategy is S, namely $g(S, *)$. Player B may choose either c or d, thus each node has two outgoing links. This graph is useful in judging the defensibility of S: This criterion concerns relative payoff differences, which are made by either unilateral cooperation or unilateral defection. Traversing every possible cycle in $g(S, *)$, therefore, we count the number of nodes with $(A_{t-1}, B_{t-1}) = (c, d)$ and subtract it from the number of nodes with $(A_{t-1}, B_{t-1}) = (d, c)$. Working with integer counts is also numerically convenient. If none of the cycles in $g(S, *)$ gives a negative value in this counting, we can say that the strategy S satisfies the defensibility criterion.

A conventional way to judge the efficiency criterion of S is to consider a transition graph of S against itself, but with error probability e. Due to error, both the players can choose either c or d, which means that each node of the transition graph has four outgoing links. One can check the corresponding stationary probability $\vec{\pi} = (\pi_0, \ldots, \pi_{63})^\intercal$, where $\intercal$ means transpose. The strategy S is efficient if π_0 converges to 100% as the error probability e approaches zero from above. The calculation of $\vec{\pi}$ can be done through linear algebraic calculation with decreasing e gradually, as has been done in Yi et al. (2017) or Murase and Baek (2018).

The above method takes into account the effects of error all at once. However, we can devise a quicker way to judge the efficiency criterion *topologically* with increasing the order of e one by one, as we will explain now. Let us begin with $g_0 = g(S, S)$ which does not take into account any error. It is a directed graph, either connected or disconnected. We write $i \to j$ if node j is reachable from i in g_0, and $i \not\to j$ otherwise. When they are mutually reachable (unreachable), we write $i \leftrightarrow j$ ($i \not\leftrightarrow j$). If a node has no outgoing links, it is called a sink. This notion is extended to a strongly connected component (SCC) as well, that is, a SCC is also called a sink if it has no outgoing links. If α is a SCC composed of nodes $\alpha_1, \alpha_2, \ldots, \alpha_s$, the stationary distribution over α is defined as $\pi_\alpha = \pi_{\alpha_1} + \pi_{\alpha_2} + \ldots + \pi_{\alpha_s}$. If a sink is reachable from a node, we say that the node is in the basin of the sink, where the basin includes the sink itself.

If $i \to 0$ and $0 \not\to i$ for every node $i \neq 0$ in g_0, the node 0 constitutes the unique sink of this graph: It is a sink, by definition. It is also unique because none of the other nodes is a sink. If this is the case, just by checking g_0 without considering error, we may conclude that the strategy S under consideration satisfies the efficiency criterion. Although we have not taken into account error-induced transitions, this conclusion can be justified in two ways: First, the detailed-balance condition implies that $\pi_i/\pi_0 \lesssim O(e)$ for every $i \neq 0$ because i can be accessed from the sink 0 only by error. Or, we can explicitly construct the derivative of the principal eigenvector by using the fact that it is non-degenerate (van der Aa et al., 2007), which implies that error-induced change in connectivity with a size of $e \ll 1$ perturbs the stationary distribution $\vec{\pi}$ by an amount of $O(e)$ at most. As $e \to 0^+$, therefore, π_0 will approach 100%.

However, if the above condition is not met, i.e., if multiple sinks coexist, it is impossible to judge the efficiency of S from g_0. We then need to take error-induced transitions into consideration. For example, let us consider g_0

with two sinks α and β, together with their respective basins $\Omega_\alpha^{(0)}$ and $\Omega_\beta^{(0)}$. In other words, every node i in this graph satisfies $i \to \alpha$ or $i \to \beta$. We define $\rho_\alpha^{(1)}$ as a set of nodes that are reachable from α via a single error, and define $\rho_\beta^{(1)}$ likewise. Formally speaking, we define $\rho_\alpha^{(k)} \equiv \{i|\alpha \underset{k}{\to} i\}$, where the subscript k below the arrow means that we are considering transitions that are mediated by k errors at least. The simple reachability relation $\alpha \to i$ is equivalent to $\alpha \underset{0}{\to} i$. We will assume that $\rho_\beta^{(1)}$ has an overlap with $\Omega_\alpha^{(0)}$ at node j, whereas $\rho_\alpha^{(1)}$ does not with $\Omega_\beta^{(0)}$. It means that $\beta \underset{1}{\to} j \to \alpha$ whereas $\alpha \underset{1}{\not\to} \beta$. Then, for every node i in $\Omega_\beta^{(0)}$, we find the following path: $i \to \beta \underset{1}{\to} j \to \alpha$. Strictly speaking, α is no longer a sink at this level of description because $\alpha \underset{1}{\to} l \in \rho_\alpha^{(1)}$ by the definition of $\rho_\alpha^{(1)}$. However, we expect that such transitions should not alter the situation significantly because $\rho_\alpha^{(1)}$ is still a subset of $\Omega_\alpha^{(0)}$, meaning that the dominant direction is $i \to \alpha$ with probability of $O(1)$. Furthermore, once the principal eigenvector $\vec{\pi}$ becomes non-degenerate due to the error-induced transition via $\rho_\beta^{(1)}$, all other perturbations $\lesssim O(e)$ that we have neglected add to $\vec{\pi}$ only small changes which vanish in the small-e limit, as can be seen from the fact that the principal eigenvector has a well-defined derivative (van der Aa et al., 2007). To summarize, this double-sink example has the following property:

$$\begin{cases} i \to \alpha \text{ and } \alpha \not\to i & \text{if } i \in \Omega_\alpha^{(0)} \\ \begin{cases} i \not\leftrightarrow \alpha \\ i \underset{1}{\to} \alpha \text{ and } \alpha \underset{1}{\not\to} i \end{cases} & \text{if } i \in \Omega_\beta^{(0)}, \end{cases} \quad (1)$$

where $\Omega_\alpha^{(0)} \cup \Omega_\beta^{(0)}$ equals the whole set of nodes by assumption, and Eq. (1) guarantees that π_α approaches 100% in the limit of $e \to 0^+$. In other words, the original basin $\Omega_\alpha^{(0)}$ has been extended to $\Omega_\alpha^{(1)} \equiv \Omega_\alpha^{(0)} \cup \Omega_\beta^{(0)}$ in the sense of Eq. (1), and π_α approaches 100% as the new basin of α coincides with the whole set of nodes. Another way to rephrase it is to construct g_1 by supplementing g_0 with additional links from γ to the member nodes of $\rho_\gamma^{(1)}$, where $\gamma \in \{\alpha, \beta\}$ denotes each sink of g_0. In terms of g_1, it can be said as follows: We have $\pi_\alpha \to 100\%$ in the small-e limit because every node i in g_1 has a certain integer $k_i \in \{0, 1\}$ such that

$$\begin{cases} i \underset{k}{\not\leftrightarrow} \alpha & \text{for } 0 \le k < k_i \\ i \underset{k}{\to} \alpha \text{ and } \alpha \underset{k}{\not\to} i & \text{for } k = k_i. \end{cases} \quad (2)$$

As a more concrete example, let us consider a Markovian system with a set of four nodes, $\{\alpha, \alpha', \alpha'', \beta\}$. The transition matrix is given as follows:

$$
W = \begin{pmatrix} 1-e & 1 & 0 & 0 \\ e & 0 & 0 & e \\ 0 & 0 & 0 & 0 \\ 0 & 0 & 1 & 1-e \end{pmatrix} \begin{matrix} \alpha \\ \alpha' \\ \alpha'' \\ \beta \end{matrix} \quad (3)
$$

$$
\begin{matrix} \alpha & \alpha' & \alpha'' & \beta \end{matrix} \quad (4)
$$

each of whose columns adds up to one. At $e = 0$, the system has two disconnected parts, $\Omega_\alpha^{(0)} = \{\alpha, \alpha'\}$ and $\Omega_\beta^{(0)} = \{\alpha'', \beta\}$. The first part with the largest eigenvalue $\lambda_\alpha(e = 0) = 1$ has the corresponding left and right eigenvectors, $\vec{y}_\alpha = (1, 1, 0, 0)$ and $\vec{x}_\alpha = (1, 0, 0, 0)^\intercal$, respectively. Likewise, the second part with the largest eigenvalue $\lambda_\beta(e = 0) = 1$ has $\vec{y}_\beta = (0, 0, 1, 1)$ and $\vec{x}_\beta = (0, 0, 0, 1)^\intercal$. Note that $\vec{y}_\alpha \cdot \vec{x}_\alpha = \vec{y}_\beta \cdot \vec{x}_\beta = 1$ and $\vec{y}_\alpha \cdot \vec{x}_\beta = \vec{y}_\beta \cdot \vec{x}_\alpha = 0$. It is a usual practice to calculate eigenvalue perturbation (Morone et al., 2016), but due to the two-fold degeneracy of this problem, we have to diagonalize the following 2×2 matrix:

$$
L = \begin{pmatrix} \vec{y}_\alpha \cdot W' \cdot \vec{x}_\alpha & \vec{y}_\alpha \cdot W' \cdot \vec{x}_\beta \\ \vec{y}_\beta \cdot W' \cdot \vec{x}_\alpha & \vec{y}_\beta \cdot W' \cdot \vec{x}_\beta \end{pmatrix} = \begin{pmatrix} 0 & 1 \\ 0 & -1 \end{pmatrix} = PDP^{-1} \quad (5)
$$

with

$$
P = \begin{pmatrix} 1 & -1 \\ 0 & 1 \end{pmatrix} \quad (6)
$$

and

$$
D = \begin{pmatrix} 0 & 0 \\ 0 & -1 \end{pmatrix}, \quad (7)
$$

where the prime denotes differentiation with respect to e. The diagonal elements of D imply that $\lambda_\alpha(e) = 1 + O(e^2)$ and $\lambda_\beta(e) = 1 - e + O(e^2)$ so that probability over $\Omega_\beta^{(0)}$ will eventually be absorbed into that over $\Omega_\alpha^{(0)}$ as soon as e becomes positive. If we look at the structures of the left and right eigenvectors, their dot products with W' in Eq. (5) clearly show that the important point is whether the error-induced transitions from a 'sink' go outside its basin. Although the first sink α survives in this example, the actual stationary distribution, $\vec{\pi} = (1/(1+e), e/(1+e), 0, 0)^\intercal$, slightly differs from $\vec{x}_\alpha = (1, 0, 0, 0)^\intercal$ because $\alpha \underset{1}{\rightarrow} \alpha'$ in Eq. (4). However, as we have already expected, the difference is insignificant in the sense that $\vec{\pi}$ converges

to $\vec{x}_\alpha$ continuously as $e \to 0$. From $\pi_{\alpha''}/\pi_{\alpha'} = 0$, we also note that transition $\alpha'' \underset{1}{\to} \alpha'$ eventually occurs with probability $e + (1-e)e + (1-e)^2 e + \ldots = 1$ in the long run because of the self-loop at β. Although it is involved with an error with probability $e \ll 1$, the total probability in the long run may be of $O(1)$, and this is what affects the stationary distribution $\vec{\pi}$. In addition, as long as the relevant connections are all preserved as described in Eq. (2), the other error-induced transitions, which may actually be involved in calculating $\vec{\pi}$, do not change the conclusion: As an example, suppose that we add to Eq. (4) transition from every node to every other with probability e^2, represented by

$$(\delta W)_{ij} = \begin{cases} -3e^2 & \text{if } i = j \\ e^2 & \text{if } i \neq j. \end{cases} \tag{8}$$

The second example is to add a transition of probability e from α' to α'' by setting $W_{\alpha''\alpha'} = e$ and $W_{\alpha\alpha'} = 1 - e$ in Eq. (4). In both of these examples, we can show by direct calculation that the resulting $\vec{\pi}$ keeps converging to $\vec{x}_\alpha$ in the small-e limit. On the other hand, if we add a transition from α to α'' with probability e, it extends $\rho_\alpha^{(1)}$ beyond $\Omega_\alpha^{(0)} = \{\alpha, \alpha'\}$, so neither α nor β survives alone but they divide up the stationary distribution in the sense that $\pi_\alpha/\pi_\beta \sim O(1)$.

Now, the above procedure can be carried out recursively:

1. Construct g_0 with a set of nodes, $\mathcal{N}$.
   - If $0 \to i$ for a certain node i, the strategy is inefficient.
   - If $\Omega_0^{(0)} \equiv \{i | i \to 0 \text{ and } 0 \not\to i\}$ equals $\mathcal{N}$, the strategy is efficient.
   - If $\Omega_0^{(0)}$ is a strict subset of $\mathcal{N}$, the efficiency criterion is undecidable from g_0. Go to the next step with $\nu = 1$.
2. Construct g_ν by adding links from every sink γ surviving in $g_{\nu-1}$ to the member nodes of $\rho_\gamma^{(\nu)}$.
   - If $0 \underset{\nu}{\to} i$ for a node i outside $\Omega_0^{(\nu-1)}$, the strategy is inefficient.
   - If $\Omega_0^{(\nu)} \equiv \Omega_0^{(\nu-1)} \cup \{i | i \underset{\nu}{\to} 0 \text{ and } 0 \underset{\nu}{\not\to} i\}$ equals $\mathcal{N}$, the strategy is efficient.
   - If $\Omega_0^{(\nu)}$ is a strict subset of $\mathcal{N}$, the efficiency criterion is undecidable from g_ν. Go to the next step.

3. Increase ν by one, and go back to the previous step.

This algorithm always ends with a decision between 'efficient' and 'inefficient' because the graph becomes strongly connected if we include all possible types of error. A pseudo-code to judge efficiency is given in Fig. 1.

The algorithm to judge the distinguishability criterion is similar to the one for the efficiency criterion as shown in the following. If S is a distinguishable strategy, the stationary probability of state $\lim_{e\to 0}\pi_0 < 1$ when players of S and AllC play the game. This is because an AllC player must have a smaller payoff than that of the co-player except at full cooperation. Although this was judged by a linear algebraic calculation in Yi et al. (2017), we can directly use the topological structure of a graph $g(S, \mathrm{AllC})$ just as in the case of the efficiency criterion. To this end, we only have to construct graphs g_ν from $g_{\nu-1}$, where g_0 is $g(S, \mathrm{AllC})$ (Fig. 2).

Exploring the memory-three strategy space

The space of memory-m strategies is represented by a complete binary tree of depth 2^{2m}. Figure 3 shows a tree of memory-one strategies. In this representation, a leaf vertex (a vertex having no child vertices) corresponds to a specific strategy whereas an internal vertex (a vertex having child vertices) represents a set of strategies, a part of whose actions remain undetermined. The root vertex of the tree corresponds to the whole set of strategies in memory-m strategy space. Traversing the tree from the root to a leaf vertex by one step is equivalent to determining one of the undetermined actions. Hereafter, this sort of tree is called a strategy tree, and the set of strategies corresponding to an internal vertex is called a strategy set. The order in which actions are determined may be arbitrary in each subtree. For example, in Fig. 3, the root vertex branches into two subtrees depending on what to do at (c, c). If the answer is c, we enter the left subtree, and the next question concerns what to do at (c, d). If we choose d at (c, c), on the other hand, we get into the right subtree, and what comes next is the choice at (d, d).

We will begin by finding strategies that satisfy the efficiency and defensibility criteria. As will be explained below, we focus on necessary conditions for a strategy set to satisfy the efficiency or defensibility criteria: If the necessary conditions are violated at an internal vertex, the whole branch below it may be discarded without further consideration. For this reason, the compu-

```
def is_efficient(strategy)
  judged = Array(64, false)
  judged[0] = true
  # => judged = [true, false, false, ..., false]

  # initialize g_n = g(S,S)
  gn = construct_g(strategy, strategy)

  until judged.all?
    64.times do |i|
      next if judged[i]
      if gn.reachable(0, i)      # 0->i
        return false             # judged as inefficient
      end
      if gn.reachable(i, 0)      # i->0 && 0!->i
        judged[i] = true
      end
    end
    gn = update_gn(gn)           # g_n <- g_{n+1}
  end
  return true
end

def update_gn(g)
  g_new = g.clone
  sink_sccs = g.sink_strongly_connected_components
  sink_sccs.each do |sink|
    for_each_node_in(sink) do |n|
      # states reachable by an error from n.
      noised_states = [n^1,n^8]
      noised_states.each do |to|
        g_new.add_link(n, to) unless g_new.has_link?(n, to)
      end
    end
  end
  return g_new
end
```

Figure 1: A pseudo-code to judge efficiency of a strategy.

```
def is_distinguishable(strategy)
  judged = Array(64, false)
  judged[0] = true
  # => judged = [true, false, false, ..., false]

  # initialize g_n = g(S, AllC)
  gn = construct_g(strategy, AllC)

  until judged.all?
    64.times do |i|
      next if judged[i]
      if gn.reachable(0, i)     # 0->i
        return true             # judged as distinguishable
      end
      if gn.reachable(i, 0)     # i->0 && 0!->i
        judged[i] = true
      end
    end
    gn = update_gn(gn)          # g_n <- g_{n+1}
  end
  return false
end
```

Figure 2: A pseudo-code to judge distinguishability of a strategy. The method 'update_gn' is identical to the one in Fig. 1.

tational cost crucially depends on in which order the actions are determined along the branches of the tree.

Checking the defensibility criterion for a strategy set

Let $g(\mathbf{S}, *)$ be the transition graph for a strategy set $\mathbf{S}$, which is defined as the largest common subgraph of $g(S, *)$ for every member strategy $S \in \mathbf{S}$, where $*$ is a wildcard character: If $g(\mathbf{S}, *)$ contains a negative cycle, $g(S, *)$ must also contain it, hence S violates defensibility. Conversely, a necessary condition for S to satisfy the defensibility criterion is the absence of negative cycles in $g(\mathbf{S}, *)$. Examples of $g(\mathbf{S}, *)$ are shown in Fig. 3. The transition graph for a strategy set is constructed in the following way: If an action at one of its nodes (i.e. history profiles) is determined, two outgoing links are added at the node. They are two because the co-player's choice can be either c or d, which leads to a different history profile at the next time step. If we have not determined the action, the node has no outgoing links.

The existence of negative cycles in $g(\mathbf{S}, *)$ is judged by the Floyd-Warshall (FW) algorithm (Hougardy, 2010). The FW algorithm finds the minimum

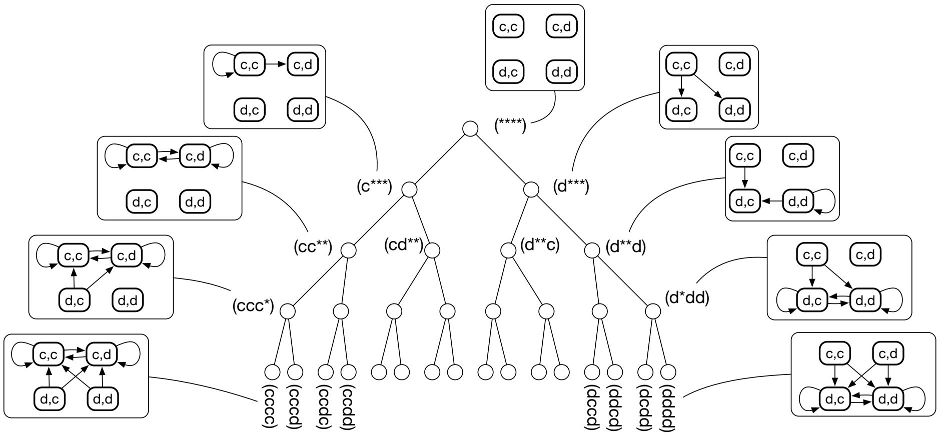

Figure 3: An example of a strategy tree of the memory-1 strategy space for the iterated PD game. A memory-1 strategy is represented by a binary string of length $2^{nm} = 4$, each of which corresponds to a leaf node of the strategy tree. The depth of the strategy tree is 4. Starting from the node, which corresponds to the strategy set whose actions are not determined at all, one of the actions is determined whenever we visit a child node. Internal nodes correspond to a strategy set. Examples of the transition graphs for strategy sets $g(\mathbf{S}, *)$ are shown as well.

distance for every pair of nodes which do not belong to a negative cycle. By distance, we mean the relative payoff difference between the players, so that outgoing links from 'positive nodes' $(**d, **c)$ and 'negative nodes' $(**c, **d)$ contribute $+1$ and -1 to the distance, respectively. The other nodes such as $(**c, **c)$ and $(**d, **d)$ contribute zero and will be called 'neutral'. Specifically, we use the following algorithm:

1. Start from the root vertex of the tree. Define δ as a 64×64 matrix of the minimum distances for all node pairs. All its elements are formally regarded as $+\infty$ at the root vertex, where no links exist yet.

2. Move to one of the child vertices, whose corresponding strategy set is denoted by $\mathbf{S}$, by determining an action at node k. This corresponds to adding two links to k, and we denote these links as $k \to u$ and $k \to v$, respectively.

   (a) Update the minimum distance between k and an arbitrary node j by calculating $\delta_{kj} = \min\{\delta_{kj}, \delta_{ku} + \delta_{uj}, \delta_{kv} + \delta_{vj})\}$.

   (b) For the every other pair of nodes i and j, update their minimum distance by calculating $\delta_{ij} = \min\{\delta_{ij}, \delta_{ik} + \delta_{kj}\}$.

   (c) If the updated matrix δ has a negative diagonal element, a negative cycle exists in $g(\mathbf{S}, *)$. Do not go deeper into this branch. Otherwise, proceed to one of the grandchild vertices recursively as in the depth-first search.

3. Check the other child vertex in the same way.

To discard strategies that are not defensible as early as possible, we should begin by checking actions that are likely to form negative cycles: If a negative node exists with undetermined actions, this should be checked first, by adding outgoing links to the node. For example, in Fig. 3, we can say that a strategy violates defensibility if it prescribes c at its unique negative node (c, d) because such prescription forms a negative cycle of $(c, d) \to (c, d) \to \ldots$. Provided that d is the correct action at (c, d), let us proceed to one of the subsequent nodes, (d, d). One must choose d here: Otherwise, we will see a negative cycle $(d, d) \to (c, d) \to (d, d) \to \ldots$. After determining these two actions, the strategy set $\mathbf{S}$ can be written as $(*d, *d)$, and it is no longer possible to form a negative cycle at this point: To revisit the negative node (c, d) to complete a cycle, one must go through the positive node (d, c). We can

say that all the possible cycles from the negative node have been *neutralized* in this strategy set $\mathbf{S} = (*d, *d)$. With just two steps, this procedure gives the list of memory-one strategies that satisfy denfensibility. In general, we will use the following procedure:

1. Determine actions at all the negative nodes, among which d must be chosen at $(\underbrace{c \cdots c}_{m}, \underbrace{d \cdots d}_{m})$ for obvious reason.

2. If we have not determined action at a node, we will call the node 'susceptible'. Let K be the set of susceptible nodes linked from the negative nodes.

3. For each $k \in K$, compute $y_k \equiv \min_{j \in G} \delta_{jk}$, where G is the set of negative nodes.

   - If k is a positive node with $y_k = -1$, remove it from K because this path is neutralized.
   - Otherwise, add two outgoing links to k by determining an action. Replace k in K by its subsequent susceptible nodes.

4. Repeat Step 3 until a negative cycle is found or K becomes empty. In the latter case, all the strategies in the remaining strategy set do not have a negative cycle.

Checking the efficiency criterion for a strategy set

Similarly, a necessary condition exists for a strategy set to satisfy the efficiency criterion. First, an efficient strategy needs to recover mutual cooperation against one-bit error at least. The transition from state 8 (ccd, ccc) and state 1 (ccc, ccd) must eventually reach state 0 in $g(S, S)$. Otherwise, it cannot be efficient. This judgement is useful for a strategy set as well: We construct a graph $g(\mathbf{S}, \mathbf{S})$ for a strategy set $\mathbf{S}$, which is defined as the largest common subgraph of $g(S, S)$ for every member strategy $S \in \mathbf{S}$ (Fig. 4). For example, if we trace the transition from state 1 or 8 in this graph and find a cycle other than 0, all the strategies in $\mathbf{S}$ cannot be efficient, and thus it is not necessary to go further than this strategy set.

The above method checks whether the mutual cooperation is tolerant against one-bit error, which is a necessary condition for efficiency. To assure

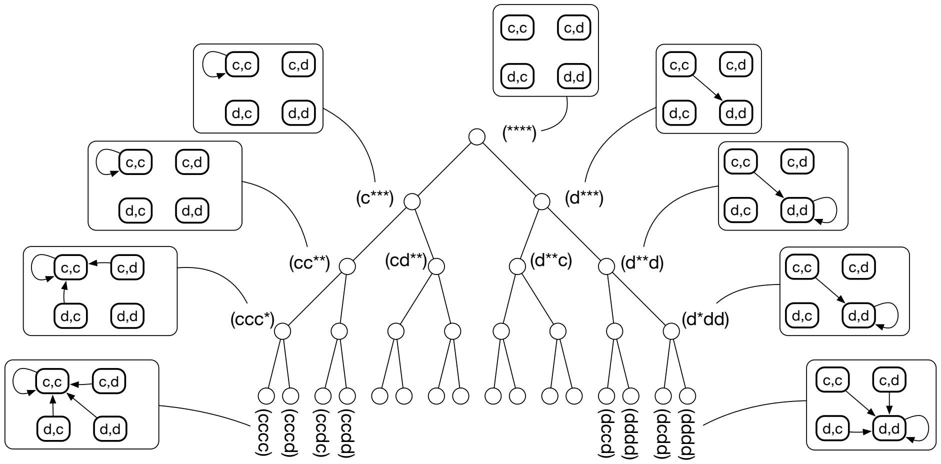

Figure 4: An example of a strategy tree of the memory-one strategy space and $g(\mathbf{S}, \mathbf{S})$, the largest common part of $g(S, S)$ for every member strategy $S \in \mathbf{S}$. Note that links may or may not be added when we go down the tree by one step. Compare this with Fig. 3 where we have depicted $g(\mathbf{S}, *)$.

the efficiency of a strategy set, we need to take into account higher-order terms of e as well. The key observation is that only SCC's can occupy finite stationary probability, which is an essential object in judging efficiency. By extending the graph-theoretic method in Fig. 1, we have developed a method to judge efficiency for a strategy set as follows: Let $\mathcal{C}$ denote the set of nodes constituting the SCC's in $g(\mathbf{S}, \mathbf{S})$. We go down the tree until every node in $\mathcal{C}$ has a prescribed action. Then, the SCC's of $g(\mathbf{S}, \mathbf{S})$ will be identical to those in $g(S, S)$ for every $S \in \mathbf{S}$. When this is the case, we use the following algorithm to test the efficiency of $\mathbf{S}$:

1. Calculate $\mathcal{C}$ for $g(\mathbf{S}, \mathbf{S})$.

2. Construct $g_0 = g(\mathbf{S}, \mathbf{S})$.

   - If $0 \to i$ for a certain node i, the strategy is inefficient.
   - If $\Omega_0^{(0)} \equiv \{i \in \mathcal{C} | i \to 0 \text{ and } 0 \not\to i\}$ equals $\mathcal{C}$, the strategy is efficient.
   - If $\Omega_0^{(0)}$ is a strict subset of $\mathcal{C}$, the efficiency criterion is undecidable from g_0. Go to the next step with $\nu = 1$.

3. Construct g_ν by adding links from every sink γ surviving in $g_{\nu-1}$ to the member nodes of $\rho_\gamma^{(\nu)}$. If g_ν has an unfixed node which is reachable from $\mathcal{C}$, fix the action at the nodes and then apply the same sequence recursively to its child strategy sets.
   - If $0 \underset{\nu}{\rightarrow} i$ for a node i outside $\Omega_0^{(\nu-1)}$, the strategy is inefficient.
   - If $\Omega_0^{(\nu)} \equiv \Omega_0^{(\nu-1)} \cup \{i \in \mathcal{C} | i \underset{\nu}{\rightarrow} 0 \text{ and } 0 \underset{\nu}{\not\rightarrow} i\}$ equals $\mathcal{C}$, the strategy is efficient.
   - If $\Omega_0^{(\nu)}$ is a strict subset of $\mathcal{C}$, the efficiency criterion is undecidable from g_ν. Go to the next step.
4. Increase ν by one, and go back to the previous step.

This algorithm always ends with a decision between 'efficient' and 'inefficient' as in the case of the algorithm for a single strategy.

Overall workflow

Another tip to reduce the number of strategies is checking the efficiency and defensibility criteria simultaneously. While the number of strategies satisfying either one of the criteria is enormous, the number is significantly reduced by checking for the efficiency and the defensibility criteria simultaneously because these two criteria require apparently contradictory behaviours (see Fig. 1 in the main text). The overall workflow is thus organized as follows:

1. Traverse the strategy tree with checking the defensibility criterion. The traversal goes down to a certain depth D_d.
2. Traverse the strategy tree with checking the efficiency criterion. The traversal goes down to D_e.
3. Repeat the above two steps with changing parameters.

If D_d or D_e is large, the number of strategy sets increases exponentially. Switching between these two steps with small depths is important to carry out the calculation in practice. We have tested various values of D_d and D_e, and found that it does not change the resulting number of successful strategies. We have also checked defensibility and efficiency of strategies that are randomly chosen from our calculation result. In short, we can say that the algorithm works as intended.

Supplementary Examples

To understand the mechanisms for successfulness in detail, we will give two examples of successful strategies, denoted by ES1 and ES2, respectively. The former is taken from the strategies having the shortest recovery path and the latter is from those with a longer recovery path.

Table 1: Action table of the first example of memory-three successful strategies, which is denoted as ES1.

	$B_{t-3}B_{t-2}B_{t-1}$							
$A_{t-3}A_{t-2}A_{t-1}$	*ccc*	*ccd*	*cdc*	*cdd*	*dcc*	*dcd*	*ddc*	*ddd*
ccc	*c*	*d*	*d*	*d*	*c*	*d*	*c*	*d*
ccd	*c*	*d*	*c*	*d*	*d*	*c*	*c*	*d*
cdc	*d*	*c*	*d*	*c*	*c*	*d*	*c*	*d*
cdd	*c*	*c*	*d*	*c*	*d*	*c*	*c*	*d*
dcc	*c*	*d*	*c*	*d*	*c*	*c*	*c*	*d*
dcd	*c*	*d*	*c*	*d*	*d*	*c*	*d*	*d*
ddc	*d*	*c*	*d*	*c*	*c*	*d*	*d*	*d*
ddd	*c*	*c*	*d*	*d*	*c*	*d*	*d*	*d*

The first example of memory-three successful strategies is defined by Table 1 and denoted as ES1. The behaviour of this strategy is distinct from TFT-ATFT in several respects: Let us look at the mechanism to stabilize the cooperation. When the players using this strategy, the mutual cooperation is recovered from a one-bit error in two steps, as we see from the first entry of Table 1 in the main text.

Although mutual cooperation is robust against a one-bit error, it does not assure that the strategy meets the efficiency criterion. This is because mutual defection is also robust against a one-bit error: When an implementation error occurs at state $63 = (ddd, ddd)$, the state eventually returns to mutual defection as

$$(ddd, ddc) \to (ddd, dcd) \to (ddd, cdd) \to (ddd, ddd). \tag{9}$$

This error robustness of mutual defection is not found in TFT-ATFT. To see how the efficiency criterion is satisfied, we need to look at higher-order transitions mediated by more than one errors. Figure 5 shows the transition

between the cycles in $g(\mathrm{ES1}, \mathrm{ES1})$. The graph has four cycles: (i) mutual cooperation (ccc, ccc), (ii) mutual defection (ddd, ddd), (iii) TFT retaliation $(cdc, dcd) \leftrightarrow (dcd, cdc)$, and (iv) synchronous repetition of cooperation and defection $(cdc, cdc) \leftrightarrow (dcd, dcd)$. The transition from mutual cooperation to mutual defection occurs with $O(e^3)$ while the transition for the opposite direction happens with $O(e^2)$. In other words, the net probability flow is towards mutual cooperation, whereby the efficiency criterion is fulfilled. This is distinct from the case for TFT-ATFT, which does not exhibit (iv). The transitions between these cycles for TFT-ATFT are also drawn in Fig. 5: Because the transition from (ii) or (iii) to (i) occurs with $O(e)$, it is sufficient to make the mutual cooperation tolerant against one-bit error to assure efficiency.

It is also instructive to convert a strategy defined by an action table to an automaton having the minimal number of states (Murase and Baek, 2020). Figure 6 shows an automaton derived from ES1, which has 15 internal states. Compared to the automaton for TFT-ATFT (Murase and Baek, 2020), it has a greater number of states with a very different graph structure. Actually, it bears more similarity to that of a successful strategy for the three-person PG game (see the dashed boxes in Fig. 6), and it is not a coincidence: Both of these two strategies have $m = 3$, and mutual cooperation is robust against two-bit errors, whereas the mutual defection is robust against one-bit error (Murase and Baek, 2018).

Another example strategy (ES2) is defined by Table 2, whose automaton representation is given in Fig. 7. Obviously, this is not a variant of the TFT-ATFT strategy, and the path to recover mutual cooperation is much longer than that of ES1 or TFT-ATFT:

$$(1,8) \to (10,17) \to (14,35) \to (16,2) \to (3,13) \to (14,19) \to \\ (16,0) \to (12,1) \to (25,1) \to (59,1) \to (19,10) \to (3,14) \to \\ (14,21) \to (21,10) \to (0,0). \quad (10)$$

Efficiency of ES2 is explained by Fig. 5 which depicts strongly connected components in $g(\mathrm{ES2}, \mathrm{ES2})$ and transition among them. Defensibility is verified by Fig. 7 because it has no negative cycle. The distinguishability criterion is also satisfied because of the cycle $14 \to 16 \to 12$, with which ES2 can repeatedly exploit an AllC player.

So far, we have focused on distinguishability only against AllC players. Generalizing this idea, we can think of a strategy that can distinguish not

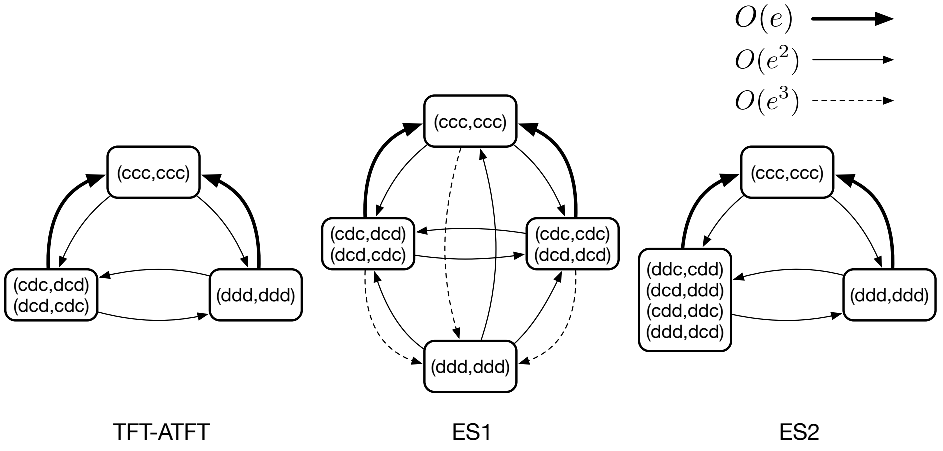

Figure 5: Transitions between strongly connected components (or cycles) in $g(S, S)$ for S =TFT-ATFT, ES1, and ES2. The thick, thin, and dashed arrows indicate transitions with probabilities $O(e)$, $O(e^2)$, and $O(e^3)$, respectively.

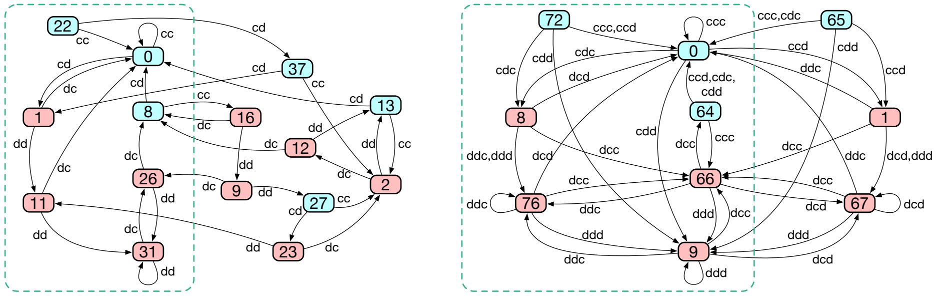

Figure 6: Automaton representations of ES1 (left) and a successful strategy for the three-person PG game (right). Colours of nodes represent prescribed actions at the corresponding history profiles: Cooperation (defection) is prescribed at blue (orange) nodes. The label on each edge means the actions taken at the last time step, $(A_{t-1}B_{t-1})$ (or $(A_{t-1}B_{t-1}C_{t-1})$ in case of the three-person game). Note the similarity between the two strategies as indicated by the dashed boxes.

only AllC but also a broader class of non-defensible strategies. Let us take WSLS as an example of non-defensible strategies. When TFT-ATFT meets WSLS, they do not achieve full cooperation, but they get the same long-term payoff when $e \to 0$, indicating that TFT-ATFT cannot distinguish a WSLS player. On the other hand, ES1 is able to distinguish a WSLS player in the sense that the long-term payoff of ES1 is strictly higher than that of WSLS, and ES1 satisfies the extended distinguishability criterion. Finally, when ES2 plays against WSLS, they form full cooperation. Thus, these three successful strategies, TFT-ATFT, ES1, and ES2, show different behaviours against WSLS.

Table 2: An action table of the second example of memory-three successful strategies (ES2).

	$B_{t-3}B_{t-2}B_{t-1}$							
$A_{t-3}A_{t-2}A_{t-1}$	*ccc*	*ccd*	*cdc*	*cdd*	*dcc*	*dcd*	*ddc*	*ddd*
ccc	*c*	*d*	*d*	*d*	*c*	*d*	*d*	*d*
ccd	*c*	*d*	*d*	*d*	*d*	*c*	*c*	*d*
cdc	*d*	*c*	*d*	*c*	*d*	*d*	*c*	*d*
cdd	*c*	*d*	*c*	*d*	*c*	*c*	*d*	*d*
dcc	*d*	*d*	*d*	*c*	*c*	*d*	*c*	*d*
dcd	*d*	*d*	*d*	*c*	*d*	*d*	*d*	*d*
ddc	*d*	*d*	*c*	*d*	*d*	*d*	*c*	*c*
ddd	*d*	*d*	*d*	*c*	*c*	*c*	*d*	*d*

When ES1 and ES2 play the game, their long-term payoffs are identical because of the defensibility criterion, but their cooperation probability is below 100%. This is because their recovery mechanisms from implementation error are different. Therefore, we can conclude that different types of successful strategies do not always achieve full cooperation although each of them meets the efficiency criterion. We have already seen many types of successful strategies in memory-three strategy space. To achieve full cooperation, players need not only adopt successful strategies but also select the same type of successful strategies. The problem thus boils down to the coordination game. In the memory-two strategy space, the situation is different because every successful variant of TFT-ATFT achieves full cooperation with every other.

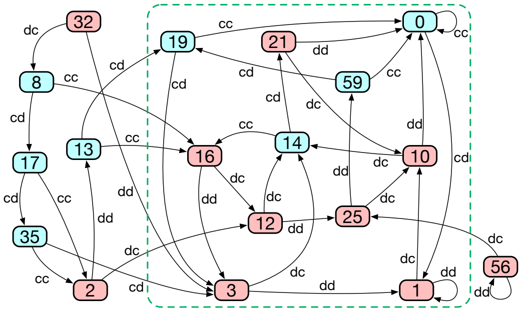

Figure 7: Automaton representation of the second example strategy (ES2). The dashed rectangle indicates the strongly connected components of the strategy.

References

Hougardy, S., 2010. The Floyd–Warshall algorithm on graphs with negative cycles. Inf. Process. Lett. 110 (8-9), 279–281.

Morone, F., Min, B., Bo, L., Mari, R., Makse, H. A., 2016. Collective influence algorithm to find influencers via optimal percolation in massively large social media. Sci. Rep. 6, 30062.

Murase, Y., Baek, S. K., 2018. Seven rules to avoid the tragedy of the commons. J. Theor. Biol. 449, 94–102.

Murase, Y., Baek, S. K., 2020. Automata representation of successful strategies for social dilemmas. Sci. Rep. 10, 13370.

van der Aa, N., Ter Morsche, H., Mattheij, R., 2007. Computation of eigenvalue and eigenvector derivatives for a general complex-valued eigensystem. Electron. J. Linear Al. 16 (1), 26.

Yi, S. D., Baek, S. K., Choi, J.-K., 2017. Combination with anti-tit-for-tat remedies problems of tit-for-tat. J. Theor. Biol. 412, 1–7.
